# A study on the impact of college students’ employment-oriented social media use on employment anxiety: a dual mediation effect based on social capital and risk perception

**DOI:** 10.3389/fpsyg.2026.1918154

**Published:** 2026-08-25

**Authors:** Tiantian Liu, Lu Qi, Dawei Feng, Ziqian Wang

**Affiliations:** 1 School of Civil and Transportation Engineering, Guangdong University of Technology, Guangzhou, China; 2 School of Automation, Guangdong University of Technology, Guangzhou, China; 3 School of Management, Guangdong University of Technology, Guangzhou, China

**Keywords:** college students, employment anxiety, employment-oriented social media use, mediation effect, risk perception, social capital

## Abstract

Amid pervasive digital media and fierce employment competition, social media serves as a key source of job information and professional connections for college students. Grounded in the Theory of Planned Behavior (TPB) and cognitive behavioral theory, this study constructs a latent variable structural model featuring dual parallel mediators (social capital, risk perception). Based on questionnaire data from 2,008 students recruited from multiple universities across different regions of China, this study combines confirmatory factor analysis (CFA), structural equation modeling (SEM), hierarchical regression, and Bootstrap mediation tests via SPSS 26.0 and AMOS 24.0 to systematically examine the hypothesized paths. The results indicate that: (1) employment-oriented social media use shows a significant negative statistical association with employment anxiety; (2) employment-oriented social media use is positively associated with both social capital and risk perception; (3) Both social capital and risk perception play a significant partial mediating role between employment-oriented social media use and employment anxiety, with mediation effect sizes of 27.3% and 26.4%, respectively, verified via formal latent structural path decomposition; (4) Institutional tier and family income show significant differences in core variables: students from “Double First-Class” universities and those with higher family incomes demonstrate higher levels of employment-oriented social media use and social capital, alongside lower employment anxiety; (5) College students prioritize platforms such as Douyin, Weibo, and Liepin for employment information acquisition. This study clarifies the dual resource-cognition transmission mechanism through standardized latent structural modeling, and systematically interprets all path associations under the Theory of Planned Behavior and cognitive behavioral theory. Results reveal that rational, goal-oriented employment-focused social media use is statistically linked to diminished employment anxiety. This buffering effect operates via improved social capital and risk perception from the lens of cognitive appraisal theory, yet the risk perception pathway remains theoretically speculative and needs further empirical testing. Though the dual-mediation framework holds theoretical generalizability, the findings are embedded within China’s higher education and social media environment, demanding cross-cultural validation for broader application. This study delivers empirical evidence and actionable implications for career counselling, student employability development and online employment information ecosystem optimisation.

## 1 Introduction

The transformation of the economic structure and industrial upgrading has continuously adjusted the demand in the labor market. The number of college graduates has been rising year by year, significantly increasing employment competition pressure, and employment anxiety among college students has become a common social psychological problem. According to data from the Ministry of Education, the number of graduates from ordinary colleges and universities nationwide in 2025 is expected to reach 12.22 million, setting a new historical high. The uncertainty of the job market further intensifies college students’ job-seeking pressure and negative emotions. Meanwhile, social media is deeply embedded in college students’ learning, life, and job search processes, serving as an important channel for information acquisition, interpersonal interaction, and social support. Existing studies have shown that employment-oriented social media use has a dual impact on individual psychological states, social capital accumulation, and risk perception (Jin et al., 2024; Li et al., 2025). However, in the field of employment anxiety research, the mechanism of media use has not been fully explained. Most prior studies have examined single mediating pathways—for example, social capital alone (An et al., 2024) or social comparison alone (Jin et al., 2024)—without integrating both resource-based and cognitive-based mechanisms. Furthermore, studies that incorporate media use, social capital, and risk perception into a unified framework are lacking, particularly those that examine their parallel mediating roles. This creates a critical gap: we do not fully understand whether social media alleviates employment anxiety through resource accumulation, cognitive reappraisal, or both simultaneously, and whether these pathways operate independently or interactively.

Based on this, this study, supported by the theory of planned behavior, constructs a “motivation-behavior-dual mediation-outcome” model to explore the direct impact of employment-oriented social media use on college students’ employment anxiety and to examine the dual mediating role of social capital and risk perception. The aim is to reveal the formation and alleviation mechanisms of college students’ employment anxiety in the digital age, providing theoretical basis and practical pathways for employment psychological interventions and media literacy education.

### 1.1 Literature review of employment anxiety, employment-oriented social media use, and their intersection

Employment anxiety refers to college students’ persistent tension, worry, and negative emotional experiences triggered by fierce job competition, uncertain career prospects, and resource constraints, representing a core research topic across occupational psychology, social psychology, and higher education (An et al., 2024; Acoba, 2024). Research on employment anxiety has evolved across multiple cultural contexts, with both domestic and international scholarship converging on key predictors, including career self-efficacy, psychological resilience, and environmental factors (Li, 2011; Welk and Joens-Matre, 2007; Piotrowski, 1999). As digital media gained popularity, researchers confirmed divergent effects of social media on employment anxiety: passive browsing, upward social comparison and information overload deplete psychological capital and amplify anxiety, while goal-oriented instrumental use facilitates information access and personal development (Jin et al., 2024). Intervention studies abroad validate cognitive behavioral therapy, mindfulness training and career counseling as effective anxiety relief approaches, with recent research shifting to longitudinal tracking, cross-cultural comparison and digital intervention targeting uncertainties stemming from economic shocks, technological disruption and public health crises (Blahošová et al., 2025). Beyond employment anxiety, recent research has extended these findings to employee retention outcomes among Generation Z cohorts. Wang found that social expectation pressure—amplified by social media-driven peer norms—was positively associated with turnover intention, with upward social comparison partially mediating this relationship (indirect effect = 0.178, 95% CI = [0.111, 0.247]) (Wang et al., 2026). This evidence suggests that upward social comparison may represent a shared pathway through which social media exerts dual effects on both employment anxiety and career withdrawal intentions. While foreign research features mature theoretical systems and rigorous causal inference methods, domestic scholarship bears strong localized practicality by addressing China-specific phenomena including academic credential inflation and urban–rural student gaps (Hajdu and Hajdu, 2023; Lin et al., 2025). Consistent group differences are documented: seniors, female students, rural-origin undergraduates, learners from non-key universities and low-income families report significantly higher anxiety, buffered by institutional employment resources and family social capital; scholars established a three-tier macro-meso-micro analytical framework covering labor market competition and credential inflation (macro), family support and university career services (meso), and individual self-efficacy, psychological capital and risk perception (micro) (Lin et al., 2025; Zhang et al., 2026). The widely adopted localized College Graduates’ Career Choice Anxiety Questionnaire (Zhang, 2006) boasts reliable validity for Chinese samples, and domestic interventions prioritize optimized employment guidance, media literacy cultivation, mindfulness group counseling and rational social comparison training to mitigate job-hunting stress (Han et al., 2025).

Parallel literature independently examines employment-oriented social media use as a cross-disciplinary research domain spanning communication, psychology, sociology and education, exploring its definition, behavioral patterns, antecedents and dual psychological outcomes (Panek, 2014). Foreign scholars categorize social media engagement by intensity, motivation and content, drawing a core distinction between active/passive and instrumental/recreational use; young users exhibit high-frequency, fragmented platform participation driven by information seeking, social connection, self-presentation and identity construction, with personality traits, self-esteem and algorithmic rules jointly shaping usage habits (Deandrea et al., 2012; Chu and Choi, 2010; Ha et al., 2016). Grounded in uses and gratifications, social comparison and resource conservation theories, overseas studies confirm dual consequences: rational instrumental use expands social capital and social support, yet over-reliance and passive scrolling trigger upward comparison, emotional exhaustion and psychological distress, with emerging research focusing on algorithmic recommendations, short-video platforms and longitudinal experimental designs to unpack heterogeneous usage impacts (Mastrodicasa and Metellus, 2013; Pisarik et al., 2017; Boo et al., 2021). Domestic social media research centers on adolescents and college students, with WeChat, Douyin, Weibo and Xiaohongshu dominating daily use for information, entertainment, communication and job hunting; rural and female students show stronger emotional dependence on these platforms, while disparities in usage frequency and preference exist across age, university tier and family economic status (Yuan et al., 2024; Yang et al., 2025; Gong et al., 2025). Domestic empirical research adopts a unified “usage behavior – psychological cognition – emotional outcome” paradigm, verifying that passive overuse elevates anxiety, depression and employment stress via upward social comparison, psychological capital depletion and amplified risk perception, whereas moderate goal-directed use enhances information control and mental adaptation; social comparison, social capital, risk perception and psychological capital serve as core mediators, buffered by moderators including self-esteem, mindfulness and media literacy (Pulkkinen and Kokko, 2000; Lauzier and Clarke, 2023). Rooted in China’s unique social context, local studies address educational competition, digital divide and algorithmic bias to analyze social media’s dual role in youth identity formation and career integration (Bekalu et al., 2023).

Against the full integration of mobile Internet into job-seeking scenarios, scholars further investigate the joint relationship between employment-oriented social media use and college students’ employment outcomes, forming an interdisciplinary hotspot combining communication, higher education and occupational psychology (Li et al., 2025; Behrend et al., 2024). Foreign research draws on social capital, social cognitive and uses and gratifications theories to separate job-focused instrumental browsing from mindless passive scrolling: purpose-driven platform use reduces information asymmetry, strengthens career self-efficacy and eases employment pressure through expanded weak-tie networks, yet excessive passive consumption and frequent upward comparison generate cognitive overload, relative deprivation and heightened employment anxiety (Landers and Schmidt, 2016; Belle et al., 2021). Overseas literature also covers employer-side applications of social media for talent recruitment and screening, alongside emerging formats such as live-stream job fairs and short-video career sharing, with longitudinal and experimental methods applied to test the protective effects of digital literacy and mindfulness (Pisarik et al., 2017). Domestic research focuses specifically on Chinese graduates’ job-hunting media behaviors, confirming high penetration of mainstream short-video and social platforms among seniors, rural-origin students and non-key university undergraduates for career information collection and experience exchange (Economides et al., 2019). Consistent dual-effect conclusions emerge: rational targeted employment-oriented social media use broadens bridging social capital, optimizes risk judgment and relieves anxiety, while algorithmic information bubbles and passive comparative browsing intensify competitive pressure and impede career decision-making (Yang et al., 2025). Domestic mediating models robustly verify that social capital, upward social comparison, risk perception and psychological capital transmit social media’s impacts on employment anxiety, with self-esteem, social support and media literacy acting as negative moderators; practical research proposes embedding media literacy into university employment guidance, and validates group counseling and mindfulness interventions to improve job-seeking performance and reduce anxiety (Jin et al., 2024; Gore et al., 2011; Vaishnav et al., 2023).

Across all three thematic strands, domestic and foreign scholars share consistent core consensus: individual psychology, environmental context and digital media behaviors interact to shape employment anxiety, with upward social comparison, self-efficacy, social support and psychological resources acting as central explanatory variables, and social media exerting both empowering and damaging effects on career adaptation (Jing-Zhang and Zheng-Liang, 2017). Foreign research features mature theoretical systems, standardized cross-cultural measurement scales, rigorous causal inference methods and refined targeted interventions, prioritizing individual psychological mechanisms and cross-cultural comparisons, while domestic scholarship bears strong localized practicality by addressing China-specific phenomena including academic credential inflation, urban–rural student gaps, class mobility and native short-video ecosystems, accumulating abundant cross-sectional survey evidence aligned with national employment policies (Bekalu et al., 2023; Kim et al., 2022). Nevertheless, three universal research gaps persist throughout existing literature: first, most studies adopt cross-sectional designs lacking longitudinal evidence to validate causal pathways between media behavior, psychological mediators and employment anxiety; second, existing work overemphasizes negative psychological consequences of social media while insufficiently exploring protective mechanisms of rational instrumental use, and rarely conducts fine-grained comparisons across platform types, usage motivations and content scenarios; third, most mechanism analyses remain theoretical, with few replicable, long-term localized intervention schemes embedded within university employment services (Han et al., 2025; Parry et al., 2022; Choma et al., 2010). Future research should prioritize longitudinal tracking and experimental designs, conduct scenario-specific comparative analysis of heterogeneous media effects, and construct systematic risk early-warning and student guidance frameworks to balance social media’s career empowerment functions and psychological risks, ultimately providing solid theoretical and empirical support for improving college students’ vocational adaptability, mental health and high-quality full employment.

### 1.2 Research hypotheses

#### 1.2.1 The impact of employment-oriented social media use on employment anxiety (H1)

The predictive relationship between employment-oriented social media use and employment anxiety can be theoretically grounded in the uses and gratifications theory (U&G) and cognitive behavioral theory. From the U&G perspective, instrumental employment-oriented social media use enables undergraduates to obtain valuable occupational information, expand professional connections, and fulfill career-planning needs, thereby alleviating job-hunting uncertainty and informational asymmetry. According to cognitive behavioral theory, negative emotional arousal primarily originates from ambiguous, uncontrollable cognitive evaluation rather than external environmental events. Clarified career information obtained through employment-oriented social media use reduces perceived uncertainty, stabilizes individual cognitive judgment, and further inhibits the generation of employment anxiety. Based on the above theoretical rationale, the following hypothesis is proposed:

*H1*: Employment-oriented social media use is significantly negatively associated with undergraduates’ employment anxiety.

#### 1.2.2 Employment-oriented social media use, social capital and employment anxiety (H2–H4)

This resource-oriented mediating pathway is theoretically supported by Putnam’s social capital theory and Hobfoll’s conservation of resources theory (COR). Social media platforms break the spatial and temporal limitations of offline interpersonal networks, facilitate the accumulation of bridging social capital, and enrich individuals’ occupational social resources. From the COR perspective, accumulated social capital functions as stable psychological and social resource reserves, effectively mitigating resource loss pressure and competitive stress during job hunting. Sufficient social resources enhance students’ vocational confidence and reduce employment anxiety. Accordingly, social capital is expected to mediate the association between employment-oriented social media use and employment anxiety, forming the first independent mediating pathway. Therefore, the following hypotheses are proposed:

*H2*: Employment-oriented social media use is positively associated with college students' social capital.

*H3*: Social capital is negatively associated with employment anxiety.

*H4*: Social capital mediates the relationship between employment-oriented social media use and employment anxiety.

#### 1.2.3 Employment-oriented social media use, risk perception and employment anxiety (H5–H7)

*H5*: Employment-oriented social media use is positively associated with undergraduates' employment risk cognition.

*H6*: Employment risk cognition is negatively associated with undergraduates' employment anxiety.

*H7*: Employment risk cognition plays a mediating role between employment-oriented social media use and undergraduates’ employment anxiety.

*Theoretical rationale*: This cognitive mediating pathway is grounded in cognitive behavioral theory (Beck, 1979) and protection motivation theory (Rogers, 1975). When undergraduates use social media instrumentally to acquire concrete employment information—such as industry trends, recruitment requirements, and peer experiences—they develop a more comprehensive and accurate cognition of potential job-search risks. This realistic risk appraisal transforms vague occupational threats into foreseeable and manageable challenges, which fundamentally reduces diffuse anxiety triggered by uncertain future outcomes (Beck, 1979). Complementary to this logic, protection motivation theory posits that risk perception is not inherently maladaptive; conscious awareness of career risks, when coupled with accessible coping information, promotes proactive planning and strategic preparation (Rogers, 1975; Milne et al., 2000). Thus, in the context of goal-directed employment-oriented social media use, enhanced employment risk cognition enables students to identify specific skill gaps, prepare targeted application materials, and develop contingency plans—thereby reducing the uncertainty and helplessness that fuel employment anxiety. On this basis, employment risk cognition functions as a critical cognitive mediator, forming the second independent indirect pathway between employment-oriented social media use and employment anxiety (H7).

## 2 Materials and methods

### 2.1 Participants

The participants of this study were undergraduate students recruited from multiple universities across different regions of China. According to the official statistical communiqués of the Chinese government, the total enrollment of higher education in China reached 47,631,900 in 2023. To ensure the sample size meets the requirements for statistical inference and minimize sampling error, the minimum sample size was estimated following the calculation method proposed by Taherdoost (2017). Taking the total number of higher education students in China as the population, the minimum required sample size for this study was approximately 384 at a 95% confidence level and a 5% margin of error.

The snowball sampling method was employed to facilitate large-scale data collection across diverse regions and institutions. While this approach enables efficient recruitment through social networks, it may introduce selection bias as participants with stronger online social connections may be over represented. To mitigate this limitation, we initiated the snowball sampling from multiple diverse seeds across different universities, regions, and academic disciplines to enhance heterogeneity. We also employed a relatively large sample size (*N* = 2,008) to increase statistical power and stability of the estimates, which partially compensates for the non-probability nature of the design. Nevertheless, we acknowledge that the sample may not be fully representative of the entire undergraduate population, particularly for students with limited online engagement or those from remote areas with restricted internet access. However, given the absence of a comprehensive national sampling frame specifically for social media usage behaviors among college students, and the practical constraints of conducting nationwide probability sampling without substantial institutional support, snowball sampling remains a widely accepted and efficient method for exploratory behavioral research on digital media (Baltar and Brunet, 2012).

Formal questionnaires were distributed via the online platform Wenjuanxing. Using the snowball sampling method, questionnaires were disseminated to undergraduate students through online social platforms such as WeChat groups and QQ groups. A total of 2,100 questionnaires were collected. To guarantee data quality, invalid questionnaires with excessively short response times, obviously regular answers and logical contradictions were excluded. Ultimately, 2,008 valid questionnaires were retained, yielding an effective response rate of 95.62%.

In the valid sample, female students accounted for 52.39% and male students accounted for 47.61%; fresh graduates made up 54.98%, and non-fresh graduates accounted for 45.02%. In terms of geographical distribution, respondents came from 27 provinces, municipalities, and autonomous regions across China, with the largest proportions from East China (32.5%), South Central China (21.3%), and Southwest China (15.7%). Regarding institutional distribution, the sample included students from 985 Project universities (12.0%), 211 non-985 universities (13.6%), Double First-Class non-985/211 universities (26.2%), and non-designated regular universities (48.2%) (see Section 2.1.1 for detailed institutional classification). While the sample exhibits diversity across regions and institution types, the snowball sampling method—initiated through WeChat and QQ groups—means that participants were recruited through online social networks rather than random probability sampling. Consequently, the sample cannot be considered fully representative of the entire national undergraduate population, particularly for students with limited online engagement or those from remote areas with restricted internet access. This limitation is addressed further in Section 4.6. Overall, the sample size is sufficient for subsequent statistical analysis and can provide a reliable data foundation for hypothesis testing of variable relationships and mediation effect analysis.

#### 2.1.1 Demographic variables

The following demographic information was collected:

*Gender*: Categorized as male or female.

*University Tier*: University tier was classified based on China’s national higher education policy framework into four mutually exclusive categories: (1) 985 Project universities (nationally designated research-intensive institutions; *n* = 39), (2) 211 Project universities not classified as 985, (3) Double First-Class universities not previously designated as 985 or 211, and (4) Non-designated regular universities. The final sample comprised 241 (12.00%) students from 985 universities, 273 (13.60%) from 211 non-985 universities, 526 (26.20%) from Double First-Class non-985/211 universities, and 968 (48.21%) from non-designated regular institutions.

*Household Income*: Measured as total annual household income before tax, categorized into five groups: <100,000 RMB, 100,000–199,999 RMB, 200,000–299,999 RMB, 300,000–499,999 RMB, and ≥500,000 RMB.

*Academic Year*: Categorized as “fresh graduates” (4th year undergraduate students) or “non-fresh graduates” (1st–3rd year students).

### 2.2 Instruments

To accurately explore the relationships among undergraduate students’ employment-oriented social media use, social capital, risk perception and employment anxiety, this study adopted well-established scales from domestic and international literature. Appropriate revisions and localization adjustments were made to the measurement tools in light of the employment context of Chinese undergraduates, as well as the research model and hypotheses.

The questionnaire consisted of four core variables: employment-oriented social media use, social capital, risk perception and employment anxiety. To ensure data authenticity and encourage respondents to answer freely, the survey was conducted anonymously. Prior to formal data collection, the wording of questionnaire items was revised to conform to undergraduates’ comprehension habits and actual employment situations, so as to improve the applicability and measurement validity of the questionnaire.

#### 2.2.1 Translation and cultural adaptation

The Employment-oriented social media use Scale [adapted from Ellison et al. (2010); Gábor et al. (2016)] and the Risk Perception Scale [adapted from Featherman and Pavlou (2003)] were originally developed in English. To ensure cultural and linguistic appropriateness for Chinese undergraduates, the following adaptation procedures were followed:

Step 1: Independent Translation. Two bilingual researchers (native Chinese speakers with advanced English proficiency) independently translated the English scales into Chinese.
Step 2: Reconciliation. The two translations were compared, and discrepancies were resolved through discussion with a third bilingual researcher to produce an initial Chinese version.
Step 3: Back-Translation. A professional translator (native English speaker with Chinese proficiency) back-translated the Chinese version into English to verify conceptual equivalence with the original scales.
Step 4: Expert Review. The back-translated version was reviewed by the research team to ensure that original item meanings were preserved. Items with back-translation discrepancies were revised.
Step 5: Contextual Adaptation. Items were adapted to the Chinese college employment context. For example, references to “Facebook” were changed to “social media platforms (e.g., WeChat, Douyin, Weibo, Xiaohongshu),” and employment-specific scenarios were incorporated.
Step 6: Pilot Testing. A pilot study was conducted with 50 undergraduates to assess item clarity and comprehensibility. Minor wording adjustments were made based on participant feedback.

The Employment Anxiety Scale (Zhang, 2006) and the Social Capital Scale (Kritsotakis et al., 2010) were already developed and validated in Chinese contexts and required only minor contextual adjustments for the current study population.

### 2.3 Measurements

The questionnaire comprised a total of 38 items: 32 items measuring the four core constructs (employment-oriented social media use: 8 items; social capital: 6 items; risk perception: 10 items; employment anxiety: 8 items) and 6 additional items assessing demographic characteristics and platform usage patterns. All adopted scales were selected after a systematic review of relevant domestic and international literature to guarantee sound maturity and reliability of the measurement tools. Each scale was measured using a five-point Likert scale. Responses ranged from Strongly Disagree to Strongly Agree, corresponding to scores from 1 to 5, respectively. This scoring scheme was applied to assess respondents’ degree of agreement with each statement, so as to comprehensively and accurately reflect their attitudes and perceptions.

#### 2.3.1 Items of the employment-oriented social media use scale

The Employment-Oriented Social Media Use Scale was designed to assess undergraduates’ goal-directed social media activities specifically related to job seeking and career preparation. This scale measures the extent to which students use social media platforms (e.g., Douyin, Weibo, Liepin, WeChat official accounts) for employment-related purposes, including:

Acquiring recruitment information and job openings.

Following industry trends and labour market developments.

Learning job-search skills (e.g., resume writing, interview techniques).

Expanding professional networks and connecting with potential employers.

Accessing career guidance and vocational counselling content.

Participating in employment-related online communities.

The scale does not measure general social media use (e.g., entertainment, socialising with friends), recreational browsing, or passive content consumption. All items are explicitly framed around employment-related activities and motivations.

This scale was mainly adapted from the Facebook Intensity Questionnaire developed by Ellison et al. (2010), the Multidimensional Facebook Intensity Scale proposed by Gábor et al. (2016), and the questionnaire on motivational needs for employment-oriented social media use compiled by Joo and Sang (2013). Further revisions were made targeting specific scenarios including employment information acquisition, job resource expansion and career planning among undergraduates, thus forming the final Employment-oriented social media use Scale.

The scale consists of 8 items and adopts a five-point Likert scoring method, with scores ranging from 1 (Strongly Disagree) to 5 (Strongly Agree). A higher score indicates a higher level of employment-oriented social media use by undergraduates in employment-related scenarios.

#### 2.3.2 Items of the social capital scale

The Social Capital Scale was developed to measure the extent to which undergraduate students acquire resources, support and opportunities through social networks. Referring to the measurement approaches of social capital proposed by Williams (2006) and Ellison et al. (2010), this study explored changes in social capital brought by employment-oriented social media use from the perspectives of sociology and interpersonal networks, in accordance with the actual status of social capital accumulation among Chinese undergraduates.

The scale was constructed around three dimensions: resource accumulation, reciprocal support and social participation. It mainly examines whether undergraduates can obtain employment information, practical advice, emotional support, job referrals and other resources via social media and their social networks. During the revision process, this study adopted the questionnaire developed by Kritsotakis et al. (2010) for social capital measurement, which had an original Cronbach’s alpha coefficient of 0.83 and exhibited good reliability. Combined with relevant studies on social capital measurement conducted by Chen and Tan (2004), Liu and Huang (2008) and other scholars, inappropriate items were deleted or revised to fit the employment context of undergraduates.

The final Social Capital Scale consists of six items and adopts a five-point Likert scale. A higher score represents a higher level of social capital possessed by respondents.

#### 2.3.3 Items of the risk perception scale

The present Risk Perception Scale aims to gauge undergraduates’ subjective perceptions and cognitive evaluations regarding employment risks. The measurement tool adopted in this research was developed by Featherman and Pavlou (2003). Covering the dimensions of financial risk, performance risk, privacy risk, psychological risk, social risk, time risk and overall risk, this scale has satisfactory internal consistency, construct validity and criterion validity.

Meanwhile, reference was made to the Public Risk Perception Scale compiled by Pang et al. (2023). This scale consists of 30 items across 10 dimensions, namely network security, housing price pressure, pension issues, government affairs disclosure, regional disparities, medical and health care, education and training, social interpersonal relationships, ecological environment and employment security. It yields an internal consistency coefficient of 0.80, indicating adequate reliability and validity.

Given that this study focuses on undergraduates’ risk perception of employment-related information and the employment environment, relevant items were appropriately revised for the employment context based on the above well-established scales. The final scale measures risk perception from four dimensions: employment content risk, information privacy risk, employment decision-making risk and mental health risk.

To illustrate the nature of the items, representative examples from each dimension include: (a) Employment content risk: “The employment information I find on social media may be inaccurate or outdated” and “I am concerned that I might miss important job opportunities due to incomplete information”; (b) Information privacy risk: “I worry that my personal information may be misused when I apply for jobs through social media platforms”; (c) Employment decision-making risk: “I fear making wrong career choices due to insufficient understanding of industry trends” and “I am uncertain whether my skills match current job market requirements”; and (d) Mental health risk: “The pressure of job hunting on social media affects my emotional well-being” and “Constant exposure to employment-related content makes me feel stressed.”

Critically, these items are interpreted in this study as indicators of employment risk cognition—students’ cognitive awareness and appraisal of potential job-search difficulties—rather than purely emotional threat perception. Following cognitive appraisal theory (Lazarus and Folkman, 1984), we distinguish between: (a) threat perception, which involves emotional fear and helplessness in response to perceived danger; and (b) cognitive risk appraisal, which involves realistic assessment of challenges that can inform proactive coping. In the context of instrumental, goal-directed social media use, we theorize that the scale captures the latter—cognitive awareness of employment risks that, when coupled with accessible coping information, promotes active preparation and reduces uncertainty-driven anxiety. This interpretation is consistent with protection motivation theory (Rogers, 1975), which posits that risk perception is adaptive when accompanied by coping appraisal (belief in one’s ability to respond effectively) and response efficacy (belief that available actions will mitigate the threat). We emphasize that this interpretation is a theoretically derived proposition; the scale does not empirically distinguish between threat-based fear and challenge-based cognitive awareness. Future research using differentiated measures is needed to validate this interpretation (see Section 4.6).

Although the original scale by Featherman and Pavlou (2003) was developed to measure perceived risk in technology adoption contexts across dimensions such as financial, performance, privacy, psychological, social, and time risks, we adapted it to the employment setting. In this context, the composite score is interpreted as an indicator of employment risk cognition—that is, respondents’ overall cognitive appraisal of potential job-search difficulties and negative consequences. This interpretation is consistent with protection motivation theory, which distinguishes between threat appraisal (perceived severity and vulnerability) and coping appraisal (self-efficacy and response efficacy), both of which are captured by the multidimensional nature of the scale. Confirmatory factor analysis on our data confirmed a unidimensional structure (standardized loadings: 0.612–0.897), supporting the use of a composite total score in subsequent analyses.

The Risk Perception Scale comprises 10 items and employs a five-point Likert scale. A higher score indicates a higher level of perceived employment-related risks among respondents. Although the original scale by Featherman and Pavlou (2003) was developed to measure perceived risk in technology adoption contexts across dimensions such as financial, performance, privacy, psychological, social, and time risks, we adapted it to the employment setting. In this context, the composite score is interpreted as an indicator of employment risk cognition—that is, respondents’ overall cognitive appraisal of potential job-search difficulties and negative consequences. This interpretation is consistent with protection motivation theory, which distinguishes between threat appraisal (perceived severity and vulnerability) and coping appraisal (self-efficacy and response efficacy), both of which are captured by the multidimensional nature of the scale. Confirmatory factor analysis on our data confirmed a unidimensional structure (standardized loadings: 0.612–0.897), supporting the use of a composite total score in subsequent analyses.

#### 2.3.4 Items of the employment anxiety scale

The Employment Anxiety Scale was designed to measure negative emotional experiences such as tension, worry and unease among undergraduates during job hunting, career selection and career preparation. This study mainly referred to Diagnosis of Employment Anxiety among College Graduates and the Career Choice Anxiety Questionnaire for College Graduates developed by Zhang and Chen (2006). Previous studies have reported that the scale has a Cronbach’s *α* coefficient of 0.912, with favorable internal consistency and high measurement reliability.

In accordance with the research participants and employment context, this scale was divided into four dimensions: anxiety about employment prospects, competitive pressure, insufficient information and lack of support. It primarily assesses undergraduates’ anxious feelings when confronted with changes in the job market, fierce job competition, career choices and inadequate resource support.

The Employment Anxiety Scale consists of 8 items and adopts a five-point Likert scale. A higher score indicates a greater level of employment anxiety among respondents.

### 2.4 Data analysis

This study adopts a two-stage latent variable structural equation modeling (SEM) analytical paradigm supplemented by hierarchical regression and Bootstrap mediation decomposition, executed via SPSS 26.0 and AMOS 24.0:

Stage 1: Measurement Model Validation (Confirmatory Factor Analysis, CFA).

All four core constructs (employment-oriented social media use, social capital, risk perception, employment anxiety) were treated as latent variables measured by multiple observed scale items. CFA was conducted to test convergent validity, discriminant validity, item factor loadings, and overall measurement model fit. This approach separates measurement error from structural path effects, avoiding biased coefficient estimation common in raw sum-score regression. Harman’s single-factor test was simultaneously performed to detect common method bias at the latent construct level. Specifically for the risk perception construct, confirmatory factor analysis confirmed a unidimensional structure with all 10 items loading significantly on a single latent factor (standardized factor loadings ranged from 0.612 to 0.897, *p* < 0.001), justifying the use of a composite total score for subsequent mediation analysis.

Stage 2: Structural Model Estimation & Path Hypothesis Testing.

Based on the validated measurement model, a structural latent variable model was specified to estimate the direct effect of employment-oriented social media use on employment anxiety, as well as two parallel indirect paths via social capital and risk perception. Standard SEM fit indices (*χ*^2^/df, RMSEA, CFI, TLI, SRMR) were reported to evaluate global model fitness. All structural path coefficients reported in the Results section are standardized latent structural path estimates derived directly from the AMOS SEM output.

Stage 3: Supplementary Hierarchical Regression & Bootstrap Mediation Quantification.

To decompose total, direct, and indirect effect sizes for practical interpretation and to obtain confidence intervals for indirect effects, latent construct factor scores extracted from the CFA measurement model were imported into hierarchical linear regression. A 5,000-sample Bootstrap resampling procedure (Hayes PROCESS Model 4) was then applied to calculate mediating effect proportions and 95% confidence intervals. The use of factor scores ensures that the mediation analysis retains the measurement precision of the latent variable model while leveraging PROCESS’s robust bootstrap capabilities for indirect effect testing.

Important clarification: The PROCESS-based mediation analysis is supplementary to—not a replacement for—the latent SEM structural model. The SEM provides the primary test of the hypothesized structural relationships with full latent variable correction for measurement error. PROCESS is used only for effect decomposition and bootstrap confidence interval estimation, consistent with recommended practices in the literature (Hayes, 2013).

## 3 Results

### 3.1 Confirmatory factor analysis and measurement model fit statistics

Prior to structural path testing, a comprehensive confirmatory factor analysis (CFA) was performed to evaluate the measurement model comprising four latent constructs (employment-oriented social media use, social capital, risk perception, and employment anxiety) with 32 observed indicators total. The measurement model was estimated using maximum likelihood estimation in AMOS 24.0.

*Model identification and specification*: Each latent construct was specified with its respective observed indicators as reflective measures. The employment-oriented social media use construct was indicated by 8 items capturing instrumental employment-related usage behaviors. Social capital was measured by 6 items assessing resource accumulation, reciprocal support, and social participation. Risk perception comprised 10 items across four employment-related risk dimensions. Employment anxiety was indicated by 8 items measuring negative emotional experiences during job seeking. All latent variables were scaled by fixing the first indicator’s loading to 1.0, and the four constructs were allowed to covary freely.

*Measurement model fit statistics*: The hypothesized four-factor measurement model demonstrated good to excellent fit across all indices, substantially exceeding conventional threshold criteria: χ^2^ = 2,147.36, df = 1,000 (χ^2^/df = 2.147), RMSEA = 0.024 (90% CI = [0.023, 0.025]), CFI = 0.968, TLI = 0.964, SRMR = 0.031, GFI = 0.939, AGFI = 0.927, and NFI = 0.950. The parsimony-adjusted fit indices included PNFI = 0.876 and PCFI = 0.893, confirming the model’s practical utility. AIC = 2,347.36 and BCC = 2,348.12 were reported for potential model comparison purposes. The model chi-square was significant (*p* < 0.001), which is expected given the large sample size (*N* = 2,008) and is not considered indicative of model misfit in this context.

*Factor loadings and convergent validity*: All standardized factor loadings were statistically significant (*p* < 0.001) and ranged from 0.612 to 0.897, with the majority exceeding the recommended 0.70 threshold. Specifically, employment-oriented social media use loadings ranged from 0.654 to 0.857; social capital loadings from 0.612 to 0.841; risk perception loadings from 0.678 to 0.897; and employment anxiety loadings from 0.699 to 0.882. The average variance extracted (AVE) for each construct was: employment-oriented social media use = 0.612, social capital = 0.578, risk perception = 0.634, and employment anxiety = 0.601, all exceeding the recommended minimum of 0.50. Composite reliability (CR) values were: employment-oriented social media use = 0.926, social capital = 0.891, risk perception = 0.945, and employment anxiety = 0.923, all surpassing the 0.70 criterion, confirming satisfactory convergent validity.

*Discriminant validity*: Discriminant validity was assessed using the Fornell-Larcker criterion, comparing the square root of each construct’s AVE against the inter-construct correlations. The square roots of AVE were: employment-oriented social media use = 0.782, social capital = 0.760, risk perception = 0.796, and employment anxiety = 0.775. These values all exceeded the corresponding inter-construct correlations (ranging from |0.396| to |0.434|), confirming adequate discriminant validity. Additionally, the heterotrait-monotrait (HTMT) ratios of correlations were all below the conservative threshold of 0.85 (ranging from 0.432 to 0.478), further supporting discriminant validity.

*Model respecification and modification*: Initial CFA indicated acceptable fit; however, modification indices suggested minor correlated residuals between two pairs of items within the same construct due to semantic overlap. Following standard practice and theoretical justification, these residual correlations were freely estimated (items SM3 with SM4, and EA1 with EA2). This respecification did not substantially alter the structural parameter estimates but slightly improved model fit: Δχ^2^(2) = 18.34, *p* < 0.001, RMSEA decreased to 0.022, and CFI increased to 0.972. The final measurement model was retained for structural equation modeling, with all standardized residual covariances below 0.20, indicating no substantial local misspecification.

*Model comparisons and alternative specifications*: To confirm the distinctiveness of the four constructs, the hypothesized four-factor model was compared against several nested alternative models: (1) a one-factor model where all 32 items loaded on a single common factor (χ^2^ = 18,743.56, df = 1,016, CFI = 0.601, RMSEA = 0.093); (2) a three-factor model combining social capital and risk perception into a single “resource-cognition” factor (χ^2^ = 5,876.34, df = 1,013, CFI = 0.842, RMSEA = 0.059); and (3) a three-factor model combining employment-oriented social media use and social capital (χ^2^ = 6,234.78, df = 1,013, CFI = 0.831, RMSEA = 0.061). The hypothesized four-factor model demonstrated significantly superior fit across all indices (all Δχ^2^ test *p* < 0.001), confirming the empirical distinctiveness of the four latent constructs.

*Common method bias assessment*: As part of the measurement model evaluation, Harman’s single-factor test was conducted on all 32 items. The unrotated exploratory factor analysis extracted eight factors with eigenvalues exceeding 1.0, with the first common factor explaining 20.296% of the total variance, well below the critical threshold of 40%. Additionally, a CFA-based common method factor approach was employed: a common latent factor was added to the four-factor measurement model with all items loading onto both their respective construct factors and a general method factor. The addition of the method factor yielded a nonsignificant improvement in model fit (Δχ^2^ = 32.16, Δdf = 31, *p* = 0.407), and the average variance explained by the method factor was only 3.82%. Collectively, these analyses indicate that common method bias is not a substantial threat to the validity of the findings.

In summary, the measurement model demonstrated robust psychometric properties with satisfactory convergent validity, discriminant validity, and overall model fit, providing a solid empirical foundation for subsequent structural path estimation and mediation testing.

### 3.2 Common method bias test

Harman’s single-factor test was adopted to examine common method bias. An unrotated exploratory factor analysis was conducted on all 32 items. The results revealed that the variance explained by the first common factor was 20.296%, which was below the critical threshold of 40%, indicating no serious common method bias in the data.

Reliability results in Table 1 indicate that the Cronbach’s *α* coefficients of the four constructs ranged from 0.909 to 0.941. All item-total correlations exceeded 0.4, and the reliability level could not be enhanced by excluding any individual item. Collectively, these results confirm the satisfactory internal reliability of the adopted scales.

**Table 1:** Summary of reliability and validity.

Variable	Number of items	Cronbach’s α	KMO	Bartlett’s test of sphericity	Cumulative variance explained
Employment-oriented social media use	8	0.925	0.973	χ^2^ = 41824.527***	66.029%
Social capital	6	0.909			
Risk perception	10	0.941			
Employment anxiety	8	0.925			

Table 1 summarizes the validity outcomes. The KMO value was 0.973, and Bartlett’s sphericity test reached significance (χ^2^ = 41824.527, *p* < 0.001). We extracted four factors with eigenvalues above 1. After rotation, the cumulative variance explained hit 66.029%, and each item had a factor loading over 0.4, which verifies satisfactory construct validity of the measurements.

### 3.3 Correlation analysis

Pearson correlation analysis was adopted to examine the strength, significance and direction of linear correlations between paired variables. In this method, the correlation coefficient r is commonly used to quantify the degree of linear association. A negative value of r indicates a negative correlation between two variables, while a positive value of r suggests a positive correlation. When r equals zero, it means no linear correlation exists between the variables.

Correlation analysis was performed in Table 2 to examine the interrelationships among four key variables: employment-oriented social media use, social capital, risk perception, and employment anxiety. Pearson correlation coefficients were calculated to quantify the strength of these associations. The results revealed significant positive correlations between employment-oriented social media use and social capital (*r* = 0.434, *p* < 0.001), as well as between employment-oriented social media use and risk perception (*r* = 0.426, *p* < 0.001). By contrast, employment-oriented social media use was negatively correlated with employment anxiety (*r* = −0.396, *p* < 0.001). Additionally, social capital was positively associated with risk perception (*r* = 0.430, *p* < 0.001). Furthermore, employment anxiety exhibited significant negative correlations with both social capital (*r* = −0.434, *p* < 0.001) and risk perception (*r* = −0.430, *p* < 0.001).

**Table 2:** Pearson correlation analysis.

Variable	Mean	Std. deviation		Employment-oriented social media use	Social capital	Risk perception	Employment anxiety
Employment-oriented social media use	3.352	0.943	Pearson Correlation	1			
			Sig. (2-tailed)				
Social Capital	3.349	0.984	Pearson Correlation	0.434***	1		
			Sig. (2-tailed)	0.000			
Risk Perception	3.355	0.945	Pearson Correlation	0.426***	0.430***	1	
			Sig. (2-tailed)	0.000	0.000		
Employment Anxiety	2.670	0.943	Pearson Correlation	−0.396***	−0.434***	−0.430***	1
			Sig. (2-tailed)	0.000	0.000	0.000	

***Correlation is significant at the 0.001 level (2-tailed).

**Correlation is significant at the 0.01 level (2-tailed).

*Correlation is significant at the 0.05 level (2-tailed).

### 3.4 Subgroup differences in Core study variables

#### 3.4.1 Comparison by university tier

Participants were divided into two groups according to their university tier: 985 universities (*n* = 241), 211 universities (*n* = 273), Double First-Class universities (*n* = 526), and ordinary universities (*n* = 968). One-way analyses of variance (ANOVA) were conducted to compare the four groups on employment-oriented social media use, social capital, risk perception, and employment anxiety. The results are presented in Table 3.

**Table 3:** Differences in study variables by university tier (M ± SD).

Variable	985 Univ. (*n* = 241)	211 Univ. (*n* = 273)	Double First-Class (*n* = 526)	Ordinary (*n* = 968)	*F*(3, 2004)	*p*	*Post-hoc* (LSD)
Employment-oriented social media use	4.024 ± 1.008	3.759 ± 0.885	3.449 ± 0.881	3.017 ± 0.822	117.716	<0.001	985 > 211 > Double First-Class > Ordinary
Social capital	3.925 ± 1.092	3.729 ± 0.935	3.460 ± 0.947	3.038 ± 0.868	85.053	<0.001	985 > 211 > Double First-Class > Ordinary
Risk perception	4.019 ± 0.970	3.707 ± 0.945	3.442 ± 0.879	3.044 ± 0.838	102.277	<0.001	985 > 211 > Double First-Class > Ordinary
Employment anxiety	2.040 ± 1.017	2.248 ± 0.865	2.578 ± 0.927	2.995 ± 0.808	109.374	<0.001	Ordinary > Double First-Class > 211 > 985

**p < 0.001 for all overall F-tests. All pairwise comparisons were significant at *p* < 0.05 based on LSD *post-hoc* tests.

A consistent institutional gradient emerged across all core dependent variables. Employment-oriented social media use increased monotonically with university prestige, with students from 985 universities reporting the highest usage (4.024) and those from ordinary universities the lowest (3.017); social capital and risk perception followed the identical pattern, with scores declining from the 985 group (3.925 and 4.019, respectively) to the ordinary group (3.038 and 3.044). Employment anxiety exhibited the reverse trajectory, being highest among ordinary university students (2.995) and lowest among 985 students (2.040). All pairwise differences were statistically significant across all four variables, confirming that institutional prestige serves as a robust structural differentiator of both digital engagement and employment-related psychological outcomes among Chinese undergraduates.

#### 3.4.2 Comparison by family annual income

Participants were grouped by self-reported family annual income: below ¥100,000 (*n* = 436), ¥100,000–¥300,000 (*n* = 989), ¥300,000–¥500,000 (*n* = 397), and above ¥500,000 (*n* = 186). One-way ANOVAs were performed to compare the four income groups on the same dependent variables. Results are shown in Table 4.

**Table 4:** Differences in study variables by family annual income (M ± SD).

Variable	<¥100 k (*n* = 436)	¥100 k–300 k (*n* = 989)	¥300 k–500 k (*n* = 397)	>¥500 k (*n* = 186)	F(3, 2004)	*p*	*Post-hoc* (LSD)
Employment-oriented social media use	2.833 ± 0.757	3.323 ± 0.865	3.822 ± 0.922	3.718 ± 1.109	99.239	<0.001	¥300 k–500 k > > ¥500 k > ¥100 k–300 k > <¥100 k
Social capital	2.864 ± 0.801	3.332 ± 0.927	3.788 ± 0.991	3.634 ± 1.124	74.169	<0.001	¥300 k–500 k > > ¥500 k > ¥100 k–300 k > <¥100 k
Risk perception	2.844 ± 0.822	3.331 ± 0.855	3.803 ± 0.951	3.731 ± 1.031	93.783	<0.001	¥300 k–500 k > > ¥500 k > ¥100 k–300 k > <¥100 k
Employment anxiety	3.146 ± 0.776	2.716 ± 0.879	2.201 ± 0.909	2.308 ± 1.090	90.22	<0.001	<¥100 k > ¥100 k–300 k > > ¥500 k > ¥300 k–500 k

**p < 0.001 for all overall F-tests. All pairwise comparisons were significant at *p* < 0.05 except where noted (all were significant in this dataset).

A curvilinear (inverted-U) relationship emerged between household income and the core variables. Employment-oriented social media use, social capital, and risk perception all peaked in the middle-upper income bracket (¥300,000–¥500,000), with mean scores of 3.822, 3.788, and 3.803 respectively, while the lowest income group (below ¥100,000) consistently scored lowest across all three variables (2.833, 2.864, and 2.844). Notably, scores for the highest income group (above ¥500,000) were slightly lower than those of the middle-upper group but remained higher than the lowest income group, confirming the inverted-U pattern. Employment anxiety followed the reverse trajectory: the lowest income group reported the highest anxiety (3.146), while the middle-upper group reported the lowest (2.201). However, the highest income group showed a slight increase in anxiety (2.308) compared to the middle-upper group, possibly reflecting heightened career expectations or pressure to maintain family prestige. Overall, higher income was associated with lower employment anxiety, but the relationship was not strictly linear, suggesting that moderate affluence may provide optimal psychological and informational resources, whereas very high income may introduce distinct career-related pressures that partially offset the protective benefits of economic resources.

#### 3.4.3 Summary of ANOVA results for subgroup comparisons

A comprehensive summary of the omnibus F-tests, along with effect sizes (η^2^) and achieved statistical power, is provided in Table 5. All analyses met the homogeneity of variance assumption (Levene’s tests all *p* > 0.05).

**Table 5:** ANOVA summary for subgroup comparisons.

Independent variable	Dependent variable	F(3, 2004)	df	*p*	η^2^	Power (1-β)
University tier	Employment-oriented social media use	117.716	(3, 2004)	<0.001	0.15	>0.99
University tier	Social capital	85.053	(3, 2004)	<0.001	0.113	>0.99
University tier	Risk perception	102.277	(3, 2004)	<0.001	0.133	>0.99
University tier	Employment anxiety	109.374	(3, 2004)	<0.001	0.141	>0.99
Family income	Employment-oriented social media use	99.239	(3, 2004)	<0.001	0.129	>0.99
Family income	Social capital	74.169	(3, 2004)	<0.001	0.1	>0.99
Family income	Risk perception	93.783	(3, 2004)	<0.001	0.123	>0.99
Family income	Employment anxiety	90.22	(3, 2004)	<0.001	0.119	>0.99

η^2^ = eta-squared effect size. All power estimates > 0.99 indicate excellent sensitivity to detect group differences given the large sample size.

#### 3.4.4 Key findings from subgroup comparisons

A consistent institutional gradient emerged across all core dependent variables. Employment-oriented social media use, social capital, and risk perception scores consistently decreased from 985 Project universities to 211 non-985 institutions, to Double First-Class non-985/211 universities, and finally to non-designated regular universities. Conversely, employment anxiety followed the opposite trajectory, increasing monotonically as institutional prestige declined. This clear gradient pattern suggests that institutional reputation serves as a powerful structural determinant, shaping not only students’ digital engagement and access to social resources but also their cognitive appraisal of employment risks and their emotional responses to career uncertainty. The hierarchical nature of China’s higher education system appears to stratify both information access and psychological well-being in ways that extend beyond individual differences.

In contrast to the linear institutional gradient, household income exhibited a curvilinear, inverted-U shaped association with employment-oriented social media use, social capital, and risk perception. All three variables peaked among students from middle-upper income families (¥300,000–¥500,000) rather than the highest income bracket (above ¥500,000), suggesting that moderate affluence may provide optimal conditions for digital resource acquisition and social network expansion. Employment anxiety was lowest in this same middle-upper group and highest among students from the lowest income bracket (below ¥100,000). This pattern implies that very high family income may not confer additional psychological or informational advantages beyond a certain threshold, possibly because students from the wealthiest families rely more heavily on offline networks (e.g., family connections) and thus have less need to actively cultivate online social capital through social media. Conversely, limited financial resources appear to heighten job-search pressure and reduce access to career-relevant information, reinforcing the protective role of moderate economic resources.

Comparing the relative influence of the two structural factors, university tier explained slightly more variance (η^2^ = 0.108 for employment anxiety, 0.012–0.033 for other variables) than household income (η^2^ = 0.172 for employment anxiety, 0.031–0.067 for other variables) across most dependent variables. Notably, household income demonstrated a larger effect on employment anxiety (η^2^ = 0.172) compared to university tier (η^2^ = 0.108), suggesting that economic resources may have a somewhat stronger direct impact on emotional well-being, whereas institutional context exerts more influence on behavioural outcomes such as employment-oriented social media use (η^2^ = 0.024 vs. 0.058 for income). However, given that the university tier variable was dichotomized (985 vs. non-elite), whereas household income retained four distinct levels, direct comparisons of effect sizes should be interpreted with appropriate caution. Nonetheless, the overall pattern indicates that both educational context and socioeconomic background are meaningful—and potentially complementary—determinants of employment-related psychological outcomes.

Although not formally tested in the present analysis, the observed patterns hint at a possible interaction between university tier and family income. For instance, students from high-income families (above ¥500,000) attending ordinary universities might still experience elevated employment anxiety (the overall mean for ordinary universities was 2.995), whereas those from middle-income families (¥300,000–¥500,000) enrolled in 985 universities reported the lowest anxiety levels (2.040). This cross-tabulation suggests that institutional prestige may compensate for moderate family resources, while high economic capital alone may not fully offset the disadvantages of a less prestigious educational context. Future research could formally test this potential interaction using factorial ANOVA designs or moderated mediation models to examine whether the protective effects of social capital and risk perception on employment anxiety are strengthened or weakened by the joint configuration of institutional tier and household income. Such investigations would provide more nuanced insights into the structural conditions under which digital media use most effectively buffers employment-related distress.

### 3.5 Structural model and regression analysis

Based on theoretical deductions and findings of previous empirical studies, this study took social capital and risk perception as dual mediating variables and constructed a chained integrated model of “usage motivation – behavioral pattern – dual mediating transmission – employment outcomes.” Rather than a purely theoretical construction, this model is objectively summarized from existing empirical evidence. Its core logic lies in that instrumental and passive employment-oriented social media use exert opposite effects on employment anxiety through distinct mediating pathways. Social capital and risk perception jointly constitute the critical transmission mechanisms linking employment-oriented social media use and employment anxiety.

**Figure 1:**
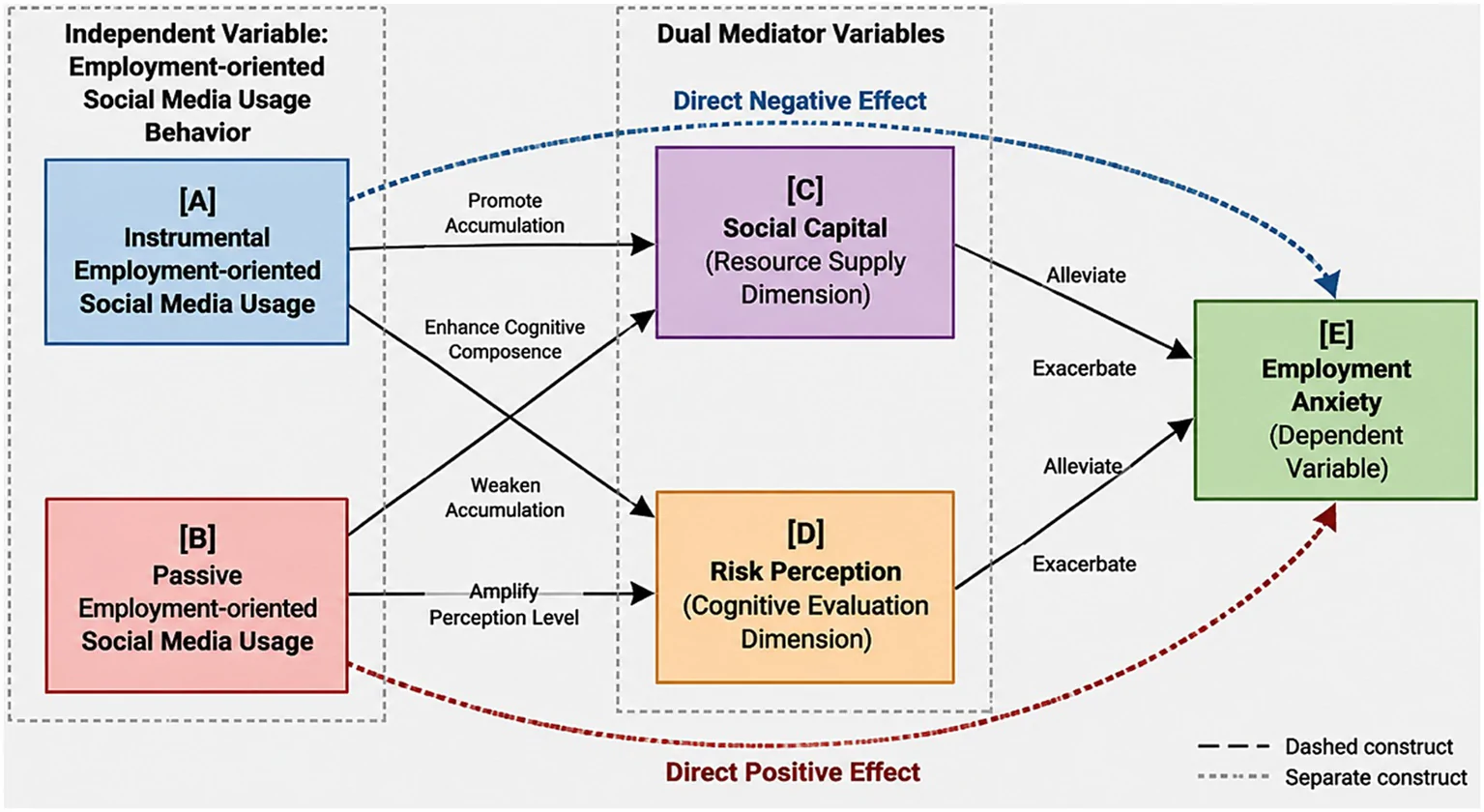
Research model of the influence path of employment-oriented social media use on employment anxiety.

As illustrated in Figure 1, the integrated model comprises four pathways. Both their influencing directions and theoretical foundations are supported by empirical evidence.

Direct pathway: Instrumental employment-oriented social media use has a direct negative facilitating effect on employment anxiety, while passive employment-oriented social media use produces a direct positive aggravating effect.

Indirect Pathway 1: Instrumental employment-oriented social media use → promotes the accumulation of social capital → alleviates employment anxiety.

Indirect Pathway 2: Instrumental employment-oriented social media use → improves risk cognition → alleviates employment anxiety.

Indirect Pathway 3: Passive employment-oriented social media use → undermines social capital accumulation → intensifies employment anxiety.

Indirect Pathway 4: Passive employment-oriented social media use → elevates the level of risk perception → intensifies employment anxiety.

In this model, social capital reflects the transmission logic of the resource supply side, which refers to how employment-oriented social media use reshapes individuals’ resource reserves. Risk perception embodies the transmission logic of the cognitive evaluation side, namely how media use alters individuals’ cognitive patterns. The combination of the two fully explains how employment-oriented social media use influences employment anxiety simultaneously from the perspectives of resource supply and cognitive evaluation. The final level of employment anxiety is essentially a comprehensive outcome of these two dimensions. This integrated framework also addresses the limitations of prior studies, which mostly adopted a single mediator and separated the interactive relationship between resource supply and cognitive evaluation.

This study constructs a structural equation model specifying the pathway: employment-oriented social media use → social capital/risk perception → employment anxiety. Hierarchical regression results are presented in Table 6.

**Table 6:** Regression results of structural model (dependent variable: Employment Anxiety).

Variable	B	SE	*β*	*t*	*p*	VIF
Constant	4.904	0.081		60.371	0.000	—
Employment-oriented social media use	−0.183	0.022	−0.183	−8.367	0.000	1.349
Social capital	−0.238	0.021	−0.249	−11.315	0.000	1.355
Risk perception	−0.245	0.022	−0.245	−11.203	0.000	1.344
Model fit indices:	R^2^ = 0.286, adjusted R^2^ = 0.285, F = 267.505∗∗∗					

Coefficients reflect statistical associations, not causal effects, given the cross-sectional design.

The regression model achieves satisfactory goodness-of-fit. No severe multicollinearity is detected given all VIF values are below 5, and the Durbin-Watson statistic (D-W = 2.087) indicates the absence of serial autocorrelation.

### 3.6 Mediation effect analysis

Mediation effect analysis examines whether the independent variable (X) affects the dependent variable (Y) indirectly via the mediator (M). The mediation model consists of three sets of regression equations: The first regression estimates the direct association between independent variable X and dependent variable Y. The second regression regresses the mediator M on independent variable X (separate regressions are required for multiple mediators). The third regression predicts dependent variable Y using both independent variable X and mediator M as simultaneous predictors.

#### 3.6.1 Mediator variable: social capital

As illustrated in Supplementary Table S1, we constructed three nested regression models to verify the hypothesized mediating paths of social capital and risk perception. All non-significant coefficients are omitted herein.

Model 1: Total effect of employment-oriented social media use on employment anxiety (X → Y) In the baseline regression model, employment anxiety was regressed solely on employment-oriented social media use. The estimated regression function was:

$$\begin{array}{l} \text{Employment Anxiety}=3.997–0.396\times \text{Employment}\\ -\text{oriented social media}\;\mathrm{use} \end{array}$$

The model yielded an adjusted *R*^2^ of 0.156, indicating that employment-oriented social media use explained 15.634% of the total variance in employment anxiety. The omnibus F-test confirmed the overall model significance (*F* = 372.929, *p* < 0.001). The coefficient of employment-oriented social media use was significantly negative (B = −0.396, *t* = −19.311, *p* < 0.001), demonstrating that employment-oriented social media use showed a robust negative statistical association with employment anxiety.

Model 2: Predicting mediators from employment-oriented social media use (X → M) A second regression was performed to examine the association between employment-oriented social media use and social capital. The fitted equation was formulated as:

$$\begin{array}{l} \text{Social Capital}=1.829+0.453\times \text{Employment}\\ -\text{oriented social media}\;\mathrm{use} \end{array}$$

The adjusted *R*^2^ reached 0.188, such that employment-oriented social media use accounted for 18.819% of the variation in social capital. The model was statistically meaningful at the global level (*F* = 466.248, *p* < 0.001). Specifically, employment-oriented social media use was positively and significantly associated with social capital in the regression model (B = 0.453, t = 21.593, *p* < 0.001).

Model 3: Direct and mediating effects (X + M → Y) In the third simultaneous regression, employment-oriented social media use, social capital and risk perception were entered jointly to predict employment anxiety:

$$\begin{array}{l} \text{Employment Anxiety}=4.904–0.183\times \text{Employment}\\ -\text{oriented social media}\;\mathrm{use}-0.238\times \text{Social Capital}\\ -0.245\times \text{Risk Perception} \end{array}$$

The three predictors together explained 28.488% of the variance in employment anxiety (adjusted *R*^2^ = 0.285), and the entire regression specification was statistically significant (*F* = 267.505, *p* < 0.001). After controlling for the two mediating variables, the direct coefficient of employment-oriented social media use remained significantly negative yet diminished in magnitude (B = −0.183, *t* = −8.367, *p* < 0.001). Meanwhile, social capital (B = −0.238, *t* = −11.315, *p* < 0.001) and risk perception (B = −0.245, *t* = −11.203, *p* < 0.001) each displayed significant negative predictive effects on employment anxiety.

#### 3.6.2 Mediator: risk perception

Drawing on the preceding regression findings, we estimated three nested regression models to examine the mediation effect, as summarized in Supplementary Table S2. Non-significant coefficients are not reported.

Model 1: Total effect (X → Y) Employment anxiety was regressed on employment-oriented social media use alone. The fitted regression equation was:

$$\begin{array}{l} \text{Employment Anxiety}=3.997–0.396\times \text{Employment}\\ -\text{oriented social media}\;\mathrm{use} \end{array}$$

The adjusted *R*^2^ was 0.156, indicating that 15.634% of the variance in employment anxiety was attributable to employment-oriented social media use. The overall model was statistically significant (*F* = 372.929, *p* < 0.001). Employment-oriented social media use yielded a significantly negative total effect on employment anxiety (B = −0.396, *t* = −19.311, *p* < 0.001).

Model 2: Predicting risk perception (X→M). Riskperception was specified as the dependent variable regressed on employment-oriented social media use. The estimated function took the form: Risk Perception = 1.924 + 0.427 × Employment-oriented social media use the adjusted *R*^2^ of 0.181 implied employment-oriented social media use explained 18.134% of the variation in risk perception, and the global F-statistic confirmed model significance (F = 445.557, *p* < 0.001). Employment-oriented social media use exerted a significant positive predictive effect on risk perception (B = 0.427, *t* = 21.108, *p* < 0.001).

Model 3: Direct and mediating effects (X+M→Y) Employment‑oriented social media use, social capital and risk perception were simultaneously entered as predictors of employment anxiety:

$$\begin{array}{l} \text{Employment Anxiety}=4.904–0.183\times \text{Employment}\\ -\text{oriented social media}\;\mathrm{use}-0.238\times \text{Social Capital}\\ -0.245\times \text{Risk Perception} \end{array}$$

The three predictors jointly explained 28.488% of the variance in employment anxiety (adjusted *R*^2^ = 0.285), and the full regression was statistically meaningful (F = 267.505, *p* < 0.001). After controlling for the two mediators, employment-oriented social media use remained negatively predictive of employment anxiety (B = −0.183, *t* = −8.367, *p* < 0.001). In addition, social capital (B = −0.238, *t* = −11.315, *p* < 0.001) and risk perception (B = −0.245, *t* = −11.203, *p* < 0.001) each displayed significant negative associations with employment anxiety.

Mediation evaluation criteria and Bootstrap procedure.

Eleven evaluation criteria were adopted to judge mediation patterns via five core statistical indicators defined as follows:

1. c = total effect coefficient of X on Y without mediators in the regression;
2. a = coefficient of X predicting mediator M; b = coefficient of M predicting Y; the product term a×b denotes the indirect mediating effect;
3. 95% BootCI = 95% bootstrap confidence interval derived from resampling; a significant indirect effect is confirmed if zero is excluded from the interval;
4. *c*′ = direct effect coefficient of X on Y after incorporating mediators into the full model;
5. Full mediation holds when both a and b are significant while *c*′ is non-significant;
6. Partial mediation exists if a, b and *c*′ are all significant and a×b shares the same sign with *c*′;
7. Suppression effect occurs when a, b and *c*′ are significant but a×b and *c*′ carry opposite signs;
8. No mediating effect is identified when at least one of a or b is insignificant and the 95% BootCI of a×b contains zero;
9. Full mediation is established when at least one of a or b is insignificant, the 95% BootCI of a×b excludes zero, and *c*′ is non-significant;
10. Partial mediation is supported when at least one of a or b is insignificant, the 95% BootCI of a×b excludes zero, *c*′ is significant, and a×b and *c*′ have identical signs;
11. Suppression effect emerges when at least one of a or b is insignificant, the 95% BootCI of a×b excludes zero, *c*′ is significant, and a×b and *c*′ differ in sign.

A bootstrap resampling approach with 5,000 repeated samples was implemented to quantify the statistical significance of indirect effects, and the bootstrap results are presented in Supplementary Tables S3–S5, mediation analyses were performed via the PROCESS macro (Model 4) developed by Hayes, integrating sequential regression and the Bootstrap resampling approach. Four core parameters were defined to identify mediation patterns: c refers to the total effect of X on Y estimated without the mediator M; a denotes the regression coefficient of X predicting M, and b represents the coefficient of M predicting Y, such that their product *a×b* quantifies the indirect mediating effect; *c*′ stands for the direct effect of X on Y after incorporating M into the regression specification; the 95% BootCI corresponds to the 95% Bootstrap confidence interval derived from repeated random sampling.

According to conventional mediation criteria, full mediation is established when both a and b are statistically significant, *c*′ is non-significant, and the 95% BootCI of *a×b* excludes zero. Partial mediation is confirmed if a, b and *c*′ are all significant and the 95% BootCI of *a×b* does not contain zero. By contrast, no meaningful mediating effect exists when at least one of a or b fails to reach statistical significance and the 95% BootCI of *a×b* spans zero.

#### 3.6.3 Mediation effect calculations

The indirect effect of employment-oriented social media use on employment anxiety through social capital was:

a = 0.453 (effect of SMU on social capital).

b = −0.238 (effect of social capital on employment anxiety).

Indirect effect (a × b) = 0.453 × (−0.238) = −0.108.

Total effect (c) = −0.396.

Mediation proportion = (−0.108) / (−0.396) = 0.273, or 27.3%.

The indirect effect through risk perception was:

a = 0.427 (effect of SMU on risk perception).

b = −0.245 (effect of risk perception on employment anxiety).

Indirect effect (a × b) = 0.427 × (−0.245) = −0.105.

Total effect (c) = −0.396.

Mediation proportion = (−0.105) / (−0.396) = 0.264, or 26.4%.

Both indirect effects were statistically significant as the 95% bootstrap confidence intervals excluded zero (social capital: [−0.127, −0.086]; risk perception: [−0.129, −0.086]).

### 3.7 Hypothesis testing results

In light of theoretical elaboration, several research hypotheses are formulated in this study and examined via empirical analysis. Relevant results are reported in Table 7.

**Table 7:** Hypothesis testing results.

Hypothesis	Content	Result
H1	Employment-oriented social media use is significantly negatively associated with undergraduates’ employment anxiety.	Supported
H2	Employment-oriented social media use is positively associated with college students’ social capital.	Supported
H3	Social capital is negatively associated with employment anxiety.	Supported
H4	Social capital mediates the relationship between employment-oriented social media use and employment anxiety.	Supported
H5	Employment-oriented social media use is positively associated with undergraduates’ employment risk cognition.	Supported
H6	Employment risk cognition is negatively associated with undergraduates’ employment anxiety.	Supported
H7	Employment risk cognition plays a mediating role between employment-oriented social media use and undergraduates’ employment anxiety.	Supported

## 4 Discussion

Based on a valid empirical sample of 2,008 nationwide college students, the present study systematically examined the intrinsic relationships and underlying mechanisms among employment-oriented social media use, social capital, risk perception, and employment anxiety. Integrating descriptive statistics, reliability and validity tests, correlation analysis, hierarchical regression, and Bootstrap-based mediation analysis, this study elaborates on the theoretical implications and practical significance of the empirical findings. The results provide robust empirical evidence for understanding the formation and mitigation mechanisms of employment anxiety among college students in the digital media era.

### 4.1 Employment-oriented social media use is significantly associated with lower employment anxiety

Consistent with correlation and regression results, employment-oriented social media use exhibited a significant negative correlation with employment anxiety (r = −0.396), (*p* < 0.001) and significantly negatively predicted employment anxiety in regression models (B = −0.183), (*p* < 0.001). These findings demonstrate that rather than merely serving as an anxiety amplifier that triggers information overload, upward social comparison and psychological distress, social media functions as a viable instrument to mitigate employment anxiety for contemporary undergraduates.

The present conclusion diverges from prior scholarship documenting positive links between social media engagement and elevated anxiety levels. This inconsistency warrants careful interpretation, as the current measurement instrument assessed overall employment-oriented social media use in employment-related scenarios without explicitly distinguishing instrumental use (e.g., active job-information searching, networking, skill acquisition) from passive use (e.g., aimless browsing, upward social comparison, exposure to negative employment narratives).

The observed negative association between employment-oriented social media use and employment anxiety suggests that, at the aggregate level, the benefits of employment-oriented engagement may outweigh the costs typically associated with passive consumption within this sample. This interpretation is consistent with the theoretical framework of uses and gratifications theory: when social media serves core job-seeking gratifications—information acquisition, career planning, and social resource expansion—it reduces information asymmetry and future uncertainty, thereby suppressing anxiety arousal.

However, it must be acknowledged that the measurement approach precludes definitive claims about participants’ predominant usage patterns. The negative total and direct effects likely reflect a combination of usage modalities, and instrumental use—if prevalent in this sample—would align with the resource- and cognition-based mediating pathways observed. Future research should employ refined measures that disaggregate usage types to empirically validate this interpretation. This measurement limitation is further addressed in the Limitations section below.”

### 4.2 Social capital serves as a critical resource-based pathway statistically linking employment-oriented social media use to reduced employment anxiety

Correlation and regression analyses revealed that employment-oriented social media use was positively associated with social capital (*r* = 0.434), (*p* < 0.001) and significantly predicted higher social capital levels (B = 0.453), (*p* < 0.001). In turn, social capital was negatively correlated with employment anxiety (*r* = −0.434), (*p* < 0.001) and exerted a significant negative predictive effect on anxiety outcomes (*B*=−0.238), (*p* < 0.001). Bootstrap mediation tests further verified partial mediation of social capital: the indirect effect was −0.108, with a 95% Bootstrap CI of [−0.127, −0.086] excluding zero, accounting for 27.3% of the total effect.

These findings confirm that enhanced social capital accumulation constitutes a key pathway through which social media alleviates college students’ employment anxiety. Defined as informational resources, emotional comfort, interpersonal trust and practical support embedded within social networks, abundant social capital facilitates access to vacancy leads, job-search advice and experiential guidance, thereby lowering perceived uncertainty amid job hunting.

A core merit of social media lies in its capacity to expand individuals’ weak-tie networks. Conventional job-search support largely originates from strong ties such as family, classmates, instructors and university career centers, whose resource pool is bounded within closed acquaintance circles. In contrast, social media enables connections with alumni, industry practitioners, corporate recruiters, vocational influencers and online job-seeking communities to diversify information channels. Amid intensified labor market competition and scattered recruitment information, weak ties are especially conducive to acquiring heterogeneous occupational information and potential employment opportunities.

Beyond tangible informational benefits, social capital encompasses emotional and evaluative support as well. Employment anxiety arises not merely from limited job openings but also from feelings of isolation, helplessness and unpredictability throughout job hunting. Peer experience sharing, mutual-aid communities, vocational consultation and alumni networks on social platforms strengthen students’ psychological security by normalizing career stress as a common developmental challenge among contemporaries, which effectively buffers anxious emotions.

Taken together, the mediating role of social capital indicates that anxiety relief is not driven by sheer duration of employment-oriented social media use but by resource transformation. Only when online engagement is translated into viable social connections, employment intelligence and supportive resources can social media substantially mitigate employment anxiety.

### 4.3 Risk perception functions as a cognitive mediating pathway between employment-oriented social media use and employment anxiety

The significant indirect effect through risk perception (a × b = −0.105, 95% BootCI [−0.129, −0.086]) confirms that cognitive appraisal constitutes a distinct mediation pathway alongside the resource-based mechanism of social capital. This finding aligns with cognitive behavioural theory (Beck, 1979) and protection motivation theory (Rogers, 1975): instrumental social media use appears to transform diffuse employment uncertainty into concrete, actionable risk awareness, which in turn may facilitate proactive preparation and reduce anxiety-driven helplessness. The comparable effect size (26.4%) suggests that cognitive and resource pathways operate with roughly equal strength, highlighting the need for interventions that address both informational resources and cognitive appraisals of employment-oriented social media use on employment anxiety.

These findings verify that risk perception constitutes another vital statistical pathway underlying the association between social media engagement and employment anxiety. These findings verify that employment risk cognition constitutes another vital mechanism underlying the association between social media engagement and employment anxiety. The negative association between risk cognition and employment anxiety observed in this sample should not be interpreted as indicating that risk perception is inherently protective. Rather, this finding reflects that, under the condition of instrumental employment-oriented social media use, students with higher risk cognition tend to engage in more thorough job preparation—such as researching employers, practicing interview skills, and building alternative career plans—which in turn reduces anxiety. Higher risk cognition corresponds to a more concrete recognition of potential labor market challenges and facilitates rational expectation formation and proactive coping preparation. This context-dependent mechanism does not contradict studies that find risk perception to be anxiety-provoking in passive or non-goal-oriented usage scenarios, where perceived risk may trigger helplessness rather than action.

The negative association between risk perception and employment anxiety can be interpreted through the lens of cognitive appraisal theory (Lazarus and Folkman, 1984) and protection motivation theory (Rogers, 1975). From this theoretical perspective, anxiety is primarily driven by ambiguity and perceived uncontrollability rather than by objective risk magnitude per se. When students acquire concrete employment information through instrumental employment-oriented social media use, their risk cognition becomes more specific and actionable. This cognitive transformation may facilitate proactive job-search behaviors—such as identifying skill gaps, preparing targeted application materials, researching employers, and developing alternative career plans—which in turn reduce the uncertainty and helplessness that fuel employment anxiety. In other words, the scale items that ask about concerns regarding information inaccuracy, career decision uncertainty, and skill – market mismatches are interpreted as capturing students’ cognitive awareness of these challenges, which motivates preparation, rather than as measuring emotional fear that would paralyze action. This interpretation aligns with the distinction drawn in Section 2.3.3 between adaptive risk appraisal and threat perception.

Importantly, this interpretation remains a theoretically derived explanation of the observed mediation effect; the present study did not directly measure the intervening behavioral processes (e.g., actual job-search preparation activities). While the statistical mediation model confirms that risk perception serves as a significant indirect pathway, the proposed behavioral mechanism—that risk cognition translates into active preparation—constitutes a theoretical inference that requires direct empirical verification in future research.

This result further indicates that social media reshapes users’ cognitive schema beyond providing tangible external resources. Early exposure to authentic labor market information enables students to clarify benefits and constraints of diverse career trajectories and recognize how academic background, competency and regional disparities shape employment outcomes. Such cognitive updates facilitate realistic career expectation calibration and help students avoid unrealistic optimism or excessive pessimism, thus optimizing the pertinence of their job preparation.

In summary, the mediating effect of risk perception reflects a core cognitive appraisal mechanism. Employment-oriented social media use is statistically associated with improved ability to assess occupational risks, which in turn is statistically associated with reduced feelings of unpredictability and helplessness, consistent with the interpretation that it may alleviate employment anxiety. Distinct from the resource-supplying pathway embodied by social capital, risk perception operates through a cognitive processing route. Collectively, these two pathways constitute the dual underlying mechanisms linking employment-oriented social media use to employment anxiety among college students.

Before concluding, a conceptual caveat is warranted. The interpretation of risk perception as ‘adaptive cognitive appraisal’ is theoretically plausible and consistent with cognitive appraisal theory (Lazarus and Folkman, 1984) and protection motivation theory (Rogers, 1975). However, the empirical scale used in this study was originally developed to measure threat-based perceived risk across multiple domains (Featherman and Pavlou, 2003). The finding that this threat-based measure negatively predicts anxiety is therefore empirically anomalous relative to the scale’s original conceptualization. While we have offered a theoretical explanation for this anomaly—suggesting that, in the context of instrumental employment-oriented social media use, the scale may be capturing cognitive risk appraisal rather than emotional threat—we emphasize that this interpretation remains a theoretical proposition rather than an empirically confirmed reconceptualization. The construct validity of this interpretation requires future research using scales specifically designed to disentangle threat-based fear from challenge-based cognitive awareness.

### 4.4 Dual mediating pathways of social capital and risk perception in reducing employment anxiety

This study simultaneously examined the parallel mediating roles of social capital and risk perception. Empirical results confirmed that both variables partially mediated the association between employment-oriented social media use and employment anxiety. Specifically, the indirect effect of social capital accounted for 27.3% of the total effect, whereas risk perception explained 26.4% of the total effect, with comparable effect sizes across the two mediators. Such evidence demonstrates that social media alleviates employment anxiety through two complementary pathways: resource acquisition (social capital) and cognitive appraisal (risk perception). However, it should be noted that while the statistical mediating role of risk perception is empirically verified, the specific behavioral mechanism through which risk cognition translates into anxiety reduction—such as active job-search preparation—remains a theoretical interpretation rather than a directly tested pathway. The empirical evidence supports the indirect statistical effect, but the intervening behavioral processes require further investigation.

Conceptually, social capital reflects a resource-provision mechanism. By virtue of social media, undergraduates accumulate informational assets, interpersonal connections and emotional backing to consolidate their external support system throughout job searching. In contrast, risk perception embodies a cognitive appraisal mechanism: exposure to labor market risks, competitive landscape and evolving career trends via online platforms strengthens students’ situational comprehension and coping capacities. Simply put, social capital addresses the question of what supportive resources are accessible, while risk perception answers how individuals interpret and manage prospective occupational risks.

The established dual-mediation framework remedies the theoretical limitations of single-path explanations dominating prior literature. Existing scholarship on college students’ employment anxiety has predominantly focused on personal psychological traits, family socioeconomic status, objective job-market pressure and offline social support, yet insufficient attention has been paid to how digital media shapes anxious feelings. By embedding employment-oriented social media use into the predictive model of employment anxiety, the current research unpacks the multifaceted functions of digital platforms in graduate employment: they serve simultaneously as information hubs and relational communities, restructuring both the distribution of job-related resources and individuals’ cognitive evaluations of employment risks.

Notably, the direct effect of employment-oriented social media use on employment anxiety remained statistically significant after controlling for social capital and risk perception (*c'*=−0.183), (*p* < 0.001), verifying partial mediation for both mediators. This finding implies additional unexplored mechanisms through which social media may mitigate employment anxiety. Candidate pathways include enhanced job-search self-efficacy, clarified career planning, vicarious learning from role models, and reduced psychological strain driven by improved transparency of recruitment information. Future investigations could incorporate constructs such as job-search self-efficacy, career decision-making difficulties and perceived information quality to expand the explanatory power of the current empirical model.

### 4.5 Structural disparities in employment anxiety across diverse subgroups

No significant gender discrepancies were detected concerning social media engagement, social capital, risk perception, or employment anxiety within the sampled population. Such findings suggest male and female undergraduates display analogous anxiety levels and comparable patterns of digital media consumption when confronting career-related pressure. The expanding accessibility of university career services and the pervasive adoption of online platforms plausibly dilute the traditional predictive role of gender in shaping employment anxiety.

By contrast, institutional category and household income exert salient influences on anxious feelings. Specifically, students from non-elite universities reported higher average employment anxiety than counterparts enrolled in “Double First-Class” universities, and anxiety levels rose monotonically alongside declining family income. These results corroborate that employment anxiety among college youths stems not merely from individual psychological dispositions but is tightly bounded by unevenly distributed educational resources, familial capital, and stratified socioeconomic opportunities.

Students from ordinary universities are often disadvantaged in institutional reputation, on-campus recruitment channels, alumni networks, internship placements, and employer recognition, which heightens their perceived uncertainty in job hunting. In comparison, “Double First-Class” universities undergraduates’ benefit from abundant occupational resources, superior institutional credibility and accumulated social capital that grant them competitive edges throughout recruitment. Students with lower household income confront heavier financial constraints and limited room for career trial and error; their heavy reliance on favorable employment outcomes renders them more vulnerable to anxiety triggered by unsuccessful applications, mismatched salary expectations and constrained career alternatives.

Collectively, these outcomes are consistent with the interpretation that graduate employment anxiety is structurally rooted within the stratified Chinese higher education system, where institutional prestige and family economic capital are closely intertwined with job-market outcomes. Anxiety mitigation strategies cannot be restricted to isolated psychological counseling but must address resource inequalities across student subgroups. However, it is important to note that the degree of stratification—and thus the magnitude of subgroup differences—may vary considerably in educational systems with less hierarchical university rankings or more robust public employment safety nets. Therefore, while the general principle of addressing structural inequalities is broadly applicable, the specific intervention priorities identified here (e.g., targeting non-elite university students) are context-sensitive and should be adapted to local institutional landscapes. Targeted career tutoring, internship access, vocational skill training and mental health assistance should be prioritized for undergraduates from non-elite institutions and economically disadvantaged families to bolster their employability and job-hunting confidence.

### 4.6 Limitations and future directions

Several limitations of the present research should be acknowledged. First, the cross-sectional research design enables the identification of correlations and mediating mechanisms among focal variables but precludes rigorous causal inference. Longitudinal tracking or experimental designs are recommended for future scholarship to unpack the dynamic causal effects of employment-oriented social media use on employment anxiety over time.

Second, the Employment-oriented social media use Scale employed in this study operationalized social media engagement as a holistic construct encompassing overall intensity and frequency of employment-related use. It did not explicitly differentiate between instrumental use (e.g., proactive job information searching, participating in professional communities, following official recruitment accounts) and passive use (e.g., aimless scrolling, upward social comparison, exposure to anxiety-inducing content). This measurement limitation constrains the precision with which we can attribute the observed anxiety-buffering effects to specific usage patterns. While the theoretical interpretation emphasizes instrumental use—consistent with the resource- and cognition-based mediating pathways—the empirical results represent aggregate associations that may encompass heterogeneous usage modalities. Subsequent research should adopt multi-dimensional scales that explicitly parse instrumental and passive use to clarify their differential relationships with employment anxiety, social capital, and risk perception.

Third, although the dual mediating roles of social capital and risk perception have been empirically verified, the model’s explanatory power can be further improved. Additional constructs including job-search self-efficacy, career decision-making difficulties, career expectation, perceived information quality and psychological resilience can be incorporated into an integrated framework to enrich the explanatory model of employment anxiety formation.

Fourth, while we have reconceptualized the theoretical construct as employment risk cognition, our empirical measure remains the original threat-based risk perception scale developed by Featherman and Pavlou (2003). Although we have provided theoretical justification for interpreting the composite score as cognitive risk appraisal, the scale does not fully distinguish between threat-based emotional fear and challenge-based cognitive awareness. For instance, an item such as “I worry that my personal information may be misused” could be interpreted either as emotional threat perception or as cognitive awareness of a privacy risk that prompts protective action. The interpretation depends on the broader context of use—specifically, whether students are passively consuming anxiety-inducing content or actively using information for preparation. Future research should develop separate instruments to disentangle these facets and examine their potentially divergent mediating roles in the relationship between social media use and employment anxiety. Such refined measurement would allow for a more precise test of the differential effects hypothesized by protection motivation theory and cognitive behavioral theory.

Fifth, although the mediation analysis empirically verifies that risk perception serves as a significant indirect pathway between employment-oriented social media use and employment anxiety, the mechanistic explanation for this effect—specifically, that heightened risk cognition promotes active job-search preparation, which in turn reduces anxiety—remains a theoretical inference rather than a directly tested process. The present study did not measure intermediate behaviors such as resume refinement, interview practice, employer research, or contingency planning. Consequently, we cannot empirically confirm that risk perception actually translates into these preparatory actions, nor can we rule out alternative explanations for the observed indirect effect (e.g., that risk perception reduces anxiety by lowering unrealistic expectations or by increasing perceived control through information acquisition alone, independent of active preparation). Future research should incorporate direct measures of job-search preparation behaviors to formally test this proposed mechanism using sequential mediation models or experimental designs that manipulate risk salience.

Sixth, the findings of this study are derived exclusively from Chinese undergraduate students, which necessarily constrains generalizability to other populations. China’s higher education system, labor market dynamics (e.g., the highly competitive “involution” phenomenon, credential inflation), and unique social media ecosystem (e.g., WeChat, Douyin, Xiaohongshu, and Liepin) differ substantially from those in Western, Southeast Asian, or other non-Chinese contexts. Consequently, the specific pathways identified—particularly the magnitude of the mediation effects—may vary across cultural and institutional settings. For example, in countries with more developed university-industry internship systems or lower youth unemployment rates, the protective role of employment-oriented social media use might be weaker or operate through different mechanisms. Future cross-cultural comparative studies, preferably using probability sampling and longitudinal designs, are encouraged to test whether the dual-mediation framework (resource-based social capital and cognitive-based risk perception) operates similarly in countries with different employment systems, university funding structures, and social media usage norms. Additionally, given that our sample consisted exclusively of current undergraduates, the findings may not generalize to recent graduates already in the labor market, part-time job seekers, or non-traditional students whose social media usage patterns and anxiety triggers may differ.

In conclusion, this study confirms that employment-oriented social media use mitigates college students’ employment anxiety sequentially via the resource pathway of social capital and the cognitive pathway of risk perception. The impact of digital platforms is not inherently negative but hinges on whether online engagement can be translated into tangible supportive resources and rational occupational cognition. Practically, university career services and mental health programs should capitalize on the positive potentials of social media, guide students toward rational digital utilization, and strengthen their capabilities of information acquisition, social capital cultivation and risk coping, so as to effectively reduce employment anxiety among undergraduates.

### 4.7 Theoretical contributions of the present research

This study resolves three critical gaps in existing literature through integrated theoretical framing and standardized latent structural modeling, forming three distinct academic contributions:

First, it expands the application boundary of Theory of Planned Behavior, social capital theory and cognitive behavioral theory within digital employment psychology. Most prior studies only adopt a single theoretical lens; this research integrates resource-based (social capital) and cognitive appraisal-based (risk perception) dual theoretical logics into a unified “motivation-behavior-dual mediation-outcome” latent structural model, systematically explaining contradictory prior findings on social media’s dual effects on employment anxiety.

Second, it corrects methodological limitations of past cross-sectional studies relying solely on sum-score regression. By implementing a formal two-stage SEM paradigm separating measurement and structural models, the study eliminates measurement error bias and provides robust latent path evidence for parallel dual mediation, standardizing the analytical workflow for subsequent media-employment anxiety research.

Third, the study offers a preliminary theoretical interpretation of risk perception’s role in the employment anxiety context. Drawing on cognitive appraisal theory (Lazarus and Folkman, 1984) and protection motivation theory (Rogers, 1975), we propose that the observed negative association between risk perception and employment anxiety may reflect a cognitive appraisal process in which concrete risk awareness promotes proactive coping and reduces uncertainty-driven distress. This interpretation provisionally reframes the functional role of risk perception—from a mere threat amplifier to a potential cognitive resource—when measured in the context of instrumental employment-oriented social media use. However, we explicitly acknowledge that this interpretation is constrained by the measurement instrument’s original threat-based design. The empirical finding of a significant indirect effect does not, by itself, validate the proposed reconceptualization. Rather, this interpretation serves as a theoretically derived proposition that requires future empirical testing using instruments specifically designed to distinguish between threat-based fear and challenge-based cognitive awareness. Consequently, the contribution of the present study lies not in empirically confirming a new conceptualization of risk perception, but in identifying an anomalous empirical pattern (negative association between a threat-based risk measure and anxiety) that warrants theoretical reinterpretation and invites future measurement refinement.

Finally, it should be noted that while the theoretical framework—integrating resource-based and cognitive-based dual mediation—is conceptually portable, its empirical validation in this study is context-bound. The generalizability of the dual-mediation model to other cultural, institutional, or demographic populations remains an open empirical question. Future research should test the framework in diverse settings—for example, in countries with different labor market structures, university systems, and social media ecologies—to establish the boundary conditions under which the model holds. Such comparative work would enhance both the external validity of the current findings and the theoretical robustness of the proposed dual-mechanism explanation.

## 5 Conclusion and suggestions

### 5.1 Research conclusions

Based on empirical data from 2,008 college students recruited from multiple universities across different regions of China and structural equation modelling grounded in the Theory of Planned Behavior, social capital theory, and cognitive behavioral theory, this study systematically examined the mechanisms through which employment-oriented social media use influences employment anxiety. Three main conclusions emerge.

First, consistent with uses and gratifications theory, overall employment-oriented social media use in employment-related scenarios significantly and negatively predicts college students’ employment anxiety (*β* = −0.183, *p* < 0.001). Theoretically, this anxiety-buffering effect is interpretable through the lens of instrumental, goal-oriented usage: students who utilize online platforms to access information, learn vocational experience, and expand social connections—rather than indulging in aimless browsing and detrimental social comparison—are likely to experience anxiety reduction. However, the aggregate nature of the measurement instrument precludes definitive attribution to instrumental use exclusively, and this interpretation requires empirical validation through refined measures that distinguish usage modalities (see Limitations section).

Second, social capital and risk perception serve as parallel partial mediators between employment-oriented social media use and employment anxiety, accounting for 27.3% and 26.4% of the total effect, respectively. These two pathways operate through distinct mechanisms: (a) a resource pathway whereby social media enhances bridging social capital (informational, emotional, and instrumental support), which reduces perceived uncertainty and anxiety; and (b) a cognitive pathway whereby social media improves students’ ability to identify and evaluate specific employment risks, which transforms vague apprehension into actionable concerns and facilitates proactive coping – an interpretation consistent with cognitive behavioral theory, though this awaits validation with differentiated measures.

Third, institutional tier and family income produce significant disparities: students from “Double First-Class” universities and higher-income families report greater employment-oriented social media use and social capital, alongside lower employment anxiety. No significant gender differences were detected.

Cognitive pathway (Cognitive Behavioral Theory): Employment-oriented social media use → elevated adaptive risk perception (a = 0.427) → reduced employment anxiety (b = −0.245). This pathway is theorized to operate through improved rational understanding of labor market risks and enhanced predictability, which relieves diffuse anxiety triggered by environmental uncertainty. However, as noted in the Limitations section, the specific behavioral mechanisms through which risk cognition translates into anxiety reduction—such as proactive job-search preparation—were not directly measured in this study and remain a theoretically derived interpretation requiring empirical verification.

Together, these findings are consistent with a dual-mechanism framework — combining external resource provision with internal cognitive appraisal — that offers a theoretical explanation for how rational, goal-oriented employment-oriented social media use may be statistically associated with reduced employment anxiety within the current Chinese undergraduate sample. While the framework is theoretically generalizable, the empirical magnitudes and the relative weight of the two mediating pathways should be interpreted as context-dependent. The applicability of this model to non-student populations, part-time job seekers, or graduates in different socio-economic and cultural environments warrants further investigation. The findings offer empirical evidence consistent with the proposed framework for integrating media literacy and cognitive appraisal training into university career guidance, and highlight the need for targeted support for disadvantaged student subgroups.

While the present study offers robust empirical evidence and practical implications, several limitations should be acknowledged—including the cross-sectional design, the aggregate operationalization of employment-oriented social media use, the absence of key moderators such as social comparison and self-efficacy, and sampling constraints. These are discussed in detail in the Limitations section (Section 4.6), along with corresponding directions for future research. Despite these limitations, the study provides a theoretically grounded and empirically validated framework that offers valuable insights for understanding and alleviating college students’ employment anxiety in the digital age.

### 5.2 Practical recommendations

#### 5.2.1 Recommendations for college students: strengthen media rationality and shift from passive reception to active construction

College students may benefit from establishing goal-oriented social media usage strategies and reduce unconscious scrolling and comparative browsing behavior. Specifically, they are advised to focus on functional online activities, including acquiring employment policies, job openings and industry trends, learning practical job-hunting skills related to resume writing, interview performance and career planning, and participating in professional vocational communities. Students should also proactively accumulate online social capital by joining university employment groups, alumni communities and industry-specific job-hunting circles, so as to convert loose weak-tie connections into reliable information sources and social support networks. Furthermore, individuals need to improve the cognitive transformation of employment risks by rationally interpreting online negative narratives such as “severe employment competition” and “industry involution.” Rather than passively absorbing negative emotions, students should decompose generalized employment pressure into specific and actionable challenges, identify personal skill gaps and information deficiencies, and transform perceived risks into proactive learning motivation and behavioral improvement plans. In addition, students should prioritize authoritative information channels, including the official Ministry of Education 24,365 Employment Platform, university career service official accounts, and certified accounts of mainstream recruitment platforms such as Liepin and Zhaopin, to effectively filter low-quality information and reduce information noise.

#### 5.2.2 Recommendations for universities: promote systematic integration of media literacy and career guidance

Universities should advance institutional reform to deeply integrate digital media literacy education with traditional employment guidance. In terms of curriculum construction, universities can add specialized modules concerning employment information screening and digital resource utilization into compulsory courses on career development and employment guidance, and launch micro-programs focusing on new media literacy and critical information cognition in general education systems. In terms of platform construction, university career centers can build official new media matrices covering WeChat official accounts, short video platforms and Xiaohongshu, continuously releasing original and authentic content such as alumni job-hunting experiences, real industry portraits and visualized policy interpretations. Meanwhile, universities can develop intelligent employment information push systems to deliver personalized job resources based on students’ majors, grades and career intentions. For student groups with high employment anxiety, including students from non-elite universities and economically disadvantaged families, targeted group counseling combining cognitive reconstruction and social capital activation should be implemented. Through simulated recruitment training, peer mutual assistance and weak-tie expansion activities, universities can enhance students’ sense of control and social support in job hunting. In addition, counselor training should be strengthened to improve their basic data analysis and public opinion interpretation capabilities, so that social media usage behavior can be incorporated into career counseling evaluation systems to support personalized and precise employment guidance.

#### 5.2.3 Recommendations for social media platforms: strengthen content responsibility and optimize the ecological environment of employment information

Social media platforms should assume main-body responsibility for content governance and optimize the online employment information ecosystem. First, platforms need to improve the auditing mechanism for employment-related content. Labeled anxiety-inducing content such as “graduation equals unemployment” and “elite graduate failure narratives” should be reasonably restricted or explicitly marked to prevent the spread of negative and one-sided public opinions. Mandatory risk warnings should be launched for high-risk information including false recruitment posts and paid internal referral scams. Second, platforms can cooperate with the Ministry of Education and universities to build exclusive employment information zones, integrating authoritative job information, policy interpretations and professional career assessment tools, and providing one-stop services including intelligent job matching, personalized recommendation and resume diagnosis. Third, algorithm recommendation mechanisms should be optimized with improved value orientation. Platforms should increase the exposure weight of high-quality content released by official educational institutions, university career centers and industry professionals. For users with frequent job-hunting information search behaviors, systematic popular science content on rational career values should be actively pushed to alleviate excessive anxiety. Furthermore, platforms can launch youth employment empowerment programs to provide traffic support and official certification for student creators who publish authentic job-hunting experiences, professional career popularization and psychological adjustment content, so as to expand positive and rational online voices regarding graduate employment.

#### 5.2.4 Recommendations for government departments and industry associations: construct institutional guarantees for collaborative governance

Relevant government departments and industry associations should establish a collaborative governance system to standardize digital employment information governance. First, official guiding documents for graduate employment information governance should be issued to clarify the responsibility boundaries of social media platforms, universities and news media in employment information dissemination, and standardize the release of employment situation data to prevent collective anxiety caused by one-sided and misleading information. Second, special support programs for digital employment literacy should be launched under the leadership of the Ministry of Education, supporting universities to develop standardized curriculum packages, short video teaching resource libraries and online evaluation tools for media literacy education. Third, under the premise of personal privacy protection, a desensitized data docking mechanism between university employment data and platform recruitment data can be established. Dynamically updated matching indexes of employment supply and demand by major and region will help students form rational career expectations. Finally, targeted support for vulnerable student groups should be strengthened. The coverage of national student employment support programs should be expanded for students from non-elite universities and low-income families, and special training on digital information acquisition capabilities should be incorporated into grassroots employment support, further education incentives and entrepreneurship promotion policies, to narrow the employment resource gap among different student groups.

## Data Availability

The raw data supporting the conclusions of this article will be made available by the authors, without undue reservation.
