# Supplementary material for: A study on the impact of college students’ employment-oriented social media use on employment anxiety: a dual mediation effect based on social capital and risk perception
Source: Front Psychol. 2026 Aug 25;17:1918154. doi: 10.3389/fpsyg.2026.1918154 (PMC13550201; doi:10.3389/fpsyg.2026.1918154)
Supplement: Supplementary file 1 [file Table_1.docx]

Supplementary Material

# Supplementary Tables

Table S1 Results of Mediation Analysis for Social Capital Pathway (n=2008)

|  | （X=>Y)Model 1  Employment Anxiety | | | | Model 2（X=>M)  **Social Capital** | | | | Model 3（X=>M=>Y)  Employment Anxiety | | | |
| --- | --- | --- | --- | --- | --- | --- | --- | --- | --- | --- | --- | --- |
|  | B | SE | t | p | B | SE | t | p | B | SE | t | p |
| **Constant** | 3.997 | 0.071 | 55.980 | 0.000 | 1.829 | 0.073 | 25.028 | 0.000 | 4.904 | 0.081 | 60.371 | < 0.001 |
| **Employment‑oriented Social Media Use** | -0.396 | 0.021 | -19.311 | 0.000 | 0.453 | 0.021 | 21.593 | 0.000 | -0.183 | 0.022 | -8.367 | < 0.001 |
| **Social Capital** |  |  |  |  |  |  |  |  | -0.238 | 0.021 | -11.315 | < 0.001 |
| Risk Perception |  |  |  |  |  |  |  |  | -0.245 | 0.022 | -11.203 | < 0.001 |
| R2 | 0.157 | | | | 0.189 | | | | 0.286 | | | |
| adjusted R2 | 0.156 | | | | 0.188 | | | | 0.285 | | | |
| F | F(1,2006)=372.929, p < 0.001 | | | | F(1,2006)=466.248, p < 0.001 | | | | F(3,2004)=267.505, p < 0.001 | | | |
| DW | 2.071 | | | | 1.916 | | | | 2.087 | | | |

Table S2 Results of Mediation Analysis for Risk Perception Pathway (n=2008)

|  | （X=>Y)Model 1  Employment Anxiety | | | | Model 2（X=>M)  **Social Capital** | | | | Model 3（X=>M=>Y)  Employment Anxiety | | | |
| --- | --- | --- | --- | --- | --- | --- | --- | --- | --- | --- | --- | --- |
|  | B | SE | t | p | B | SE | t | p | B | SE | t | p |
| **Constant** | 3.997 | 0.071 | 55.980 | 0.000 | 1.924 | 0.070 | 27.307 | 0.000 | 4.904 | 0.081 | 60.371 | < 0.001 |
| **Employment‑oriented Social Media Use** | -0.396 | 0.021 | -19.311 | 0.000 | 0.427 | 0.020 | 21.108 | 0.000 | -0.183 | 0.022 | -8.367 | < 0.001 |
| **Social Capital** |  |  |  |  |  |  |  |  | -0.238 | 0.021 | -11.315 | < 0.001 |
| Risk Perception |  |  |  |  |  |  |  |  | -0.245 | 0.022 | -11.203 | < 0.001 |
| R2 | 0.157 | | | | 0.182 | | | | 0.286 | | | |
| adjusted R2 | 0.156 | | | | 0.181 | | | | 0.285 | | | |
| F | F(1,2006)=372.929, p < 0.001 | | | | F(1,2006)=445.557, p < 0.001 | | | | F(3,2004)=267.505, p < 0.001 | | | |
| DW | 2.071 | | | | 2.021 | | | | 2.087 | | | |

Table S3 Summary of Mediation Test Results (***p < 0.001, p < 0.01, *p < 0.05)

| Pathway | c | a | b | a*b | a*b | a*b | c’ | c’ | Conclusion |
| --- | --- | --- | --- | --- | --- | --- | --- | --- | --- |
|  | Total Effect |  |  | Indirect Effect | (Boot SE) | (95% BootCI) | Direct Effect | (95% CI) |  |
| **Employment‑oriented** Social Media Use → Social Capital → Employment Anxiety | -0.396*** | 0.453*** | -0.238*** | -0.108*** | 0.010 | [–0.127, –0.086] | -0.183*** | [-0.227,-0.140] | Partial Mediation |

Table S4 Summary of Mediation Effect Size Results（***p < 0.001, p < 0.01, *p < 0.05）

| Path | Conclusion | c | a*b | c’ | Formula of Effect Proportion | Proportion of Mediated Effect |
| --- | --- | --- | --- | --- | --- | --- |
|  |  | Total Effect | Indirect Effect | Direct Effect |  |  |
| **Employment‑oriented** Social Media Use → Social Capital → Employment Anxiety | Partial Mediation | -0.396*** | -0.108*** | -0.183*** | a * b / c | 0.273 |

Note: c = total effect; a×b = indirect (mediating) effect computed as the product of path coefficients a and b; c′ = direct effect.

Table S5 Summary of Mediation Test Results（***p < 0.001, p < 0.01, *p < 0.05）

| Pathway | c | a | b | a*b | a*b | a*b | c’ | c’ | Conclusion |
| --- | --- | --- | --- | --- | --- | --- | --- | --- | --- |
|  | Total Effect |  |  | Indirect Effect | (Boot SE) | (95% BootCI) | Direct Effect | (95% CI) |  |
| Social Media Use → Risk Perception → Employment Anxiety | -0.396*** | 0.427*** | -0.245*** | -0.105*** | 0.011 | [-0.129,-0.086] | -0.183*** | [-0.227,-0.140] | Partial Mediation |

Table S6 Summary of Mediation Effect Size Results（***p < 0.001, p < 0.01, *p < 0.05）

| Pathway | Conclusion | c | a*b | c’ | Formula of Effect Proportion | Proportion of Mediated Effect |
| --- | --- | --- | --- | --- | --- | --- |
|  |  | Total Effect | Indirect Effect | Direct Effect |  |  |
| Social Media Use → Risk Perception → Employment Anxiety | Partial Mediation | -0.396*** | -0.105*** | -0.183*** | a * b / c | 0.264 |

Note: c = total effect; $\text{a}\text{×}\text{b}$ = indirect (mediating) effect calculated as the product of coefficients a and b; $\text{c}\text{'}$ = direct effect.
